# Supplementary material for: Early Detection of Apathetic Phenotypes in Huntington’s Disease Knock-in Mice Using Open Source Tools
Source: Sci Rep. 2018 Feb 2;8:2304. doi: 10.1038/s41598-018-20607-7 (PMC5797185; doi:10.1038/s41598-018-20607-7)
Supplement: Supplementary file 1 — Supplementary information [file 41598_2018_20607_MOESM1_ESM.pdf]

Supplemental Material

Early Detection of Apathetic Phenotypes in Huntington's Disease Knock-in Mice Using Open Source Tools

Shawn Minnig [1,**], Robert M. Bragg [1,**], Hardeep S. Tiwana [1], Wes T. Solem [1], William S. Hovander [1], Eva-Mari S. Vik [1], Madeline Hamilton [1], Samuel R.W. Legg, Dominic D. Shuttleworth, Sydney R. Coffey [1], Jeffrey P. Cantle [1], Jeffrey B. Carroll [1,*]

[1] Behavioral Neuroscience Program, Department of Psychology, Western Washington University, Bellingham WA 98225

** Equal Contribution

* Corresponding author:

Affiliations:

Behavioral Neuroscience Program, Department of Psychology, Western Washington University, Bellingham WA 98225

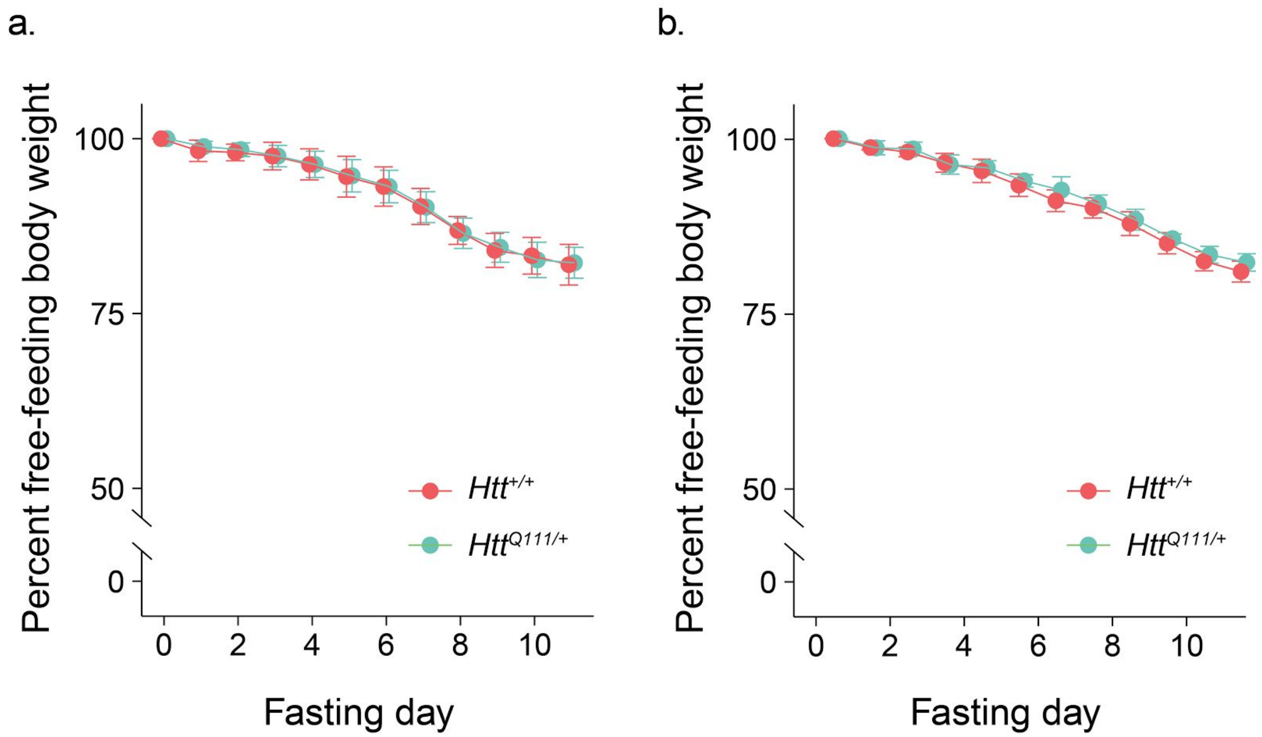

Supplemental Fig 1: Body weight during food restriction of 10-month-old *Htt*$^{+/+}$ and *Htt*$^{Q111/+}$ mice. Body weight was reduced during 11 days of food restriction, but genotype did not change overall body weight, or the rate at which mice lost weight . **a.** Initial cohort: linear mixed effects model, effect of date $F_{(11,\ 187)}$ = 673.6, p < 0.0001; effect of genotype $F_{(1,\ 17)}$ < 0.001, p = 0.96; genotype x date interaction $F_{(11,\ 187)}$ = 0.5, p = 0.87). **b.** Replication cohort: linear mixed effects model, effect of date $F_{(11,\ 242)}$ = 889.9, p < 0.0001, effect of genotype $F_{(1,\ 22)}$ = 2.6, p = 0.12, effect of genotype x date interaction $F_{(11,\ 242)}$ = 1.33, p = 0.21. Error bars represent S.E.M.
